# Ultraviolet light screen using cholesteric liquid crystal capsules on the basis of selective reflection†

**DOI:** 10.1039/d1ra03499e

**Published:** 2021-07-22

**Authors:** Heemuk Oh, Hirotugu Kikuchi, Ji Hyun Lee, Su Ji Kim, Jun Bae Lee, Moon Sun Cho, Min Young Lee, Yasushi Okumura, Joo-Hee Hong, Sung-Kyu Hong

**Affiliations:** Department of Chemical & Biochemical Engineering, Dongguk University-Seoul Seoul 100-715 Republic of Korea hsk5457@dongguk.edu; Institute for Materials Chemistry and Engineering, Kyushu University Fukuoka 816-8580 Japan; Cosmax Inc. Seongnam 13486 Republic of Korea; Department of Industrial Chemical Engineering, Suncheon Jeil College 17 Jeildaehak-gil Suncheon Jeonnam 540-744 Republic of Korea

## Abstract

Sunscreen can protect human skin from sunlight by decreasing exposure to ultraviolet (UV) light, specifically UV-B and UV-A. In this study, a new type of UV screen system is proposed using cholesteric liquid crystal (CLC) capable of selectively reflecting UV-A within the human skin temperature range of 32–36 °C. Polycaprolactone (PCL) capsules with CLC mixture which had a helical chiral pitch corresponding to the wavelength of UV light were made by a solvent evaporation method. The average diameter of the capsules was about 34 μm. Consequently, it was confirmed that the CLC mixture (COC : CN = 80 : 20) could reflect UV-A light over 350–380 nm within the human skin temperature range. Also, it was confirmed that the CLC/PCL microcapsules could block UV light over 290–400 nm by about 6%.

## Abstract 2

When the prepared cholesteric liquid crystal microcapsule is applied to the skin, it can protect the skin by selectively reflecting only ultraviolet rays in sunlight like sunscreen cosmetics.

## Graphical abstract

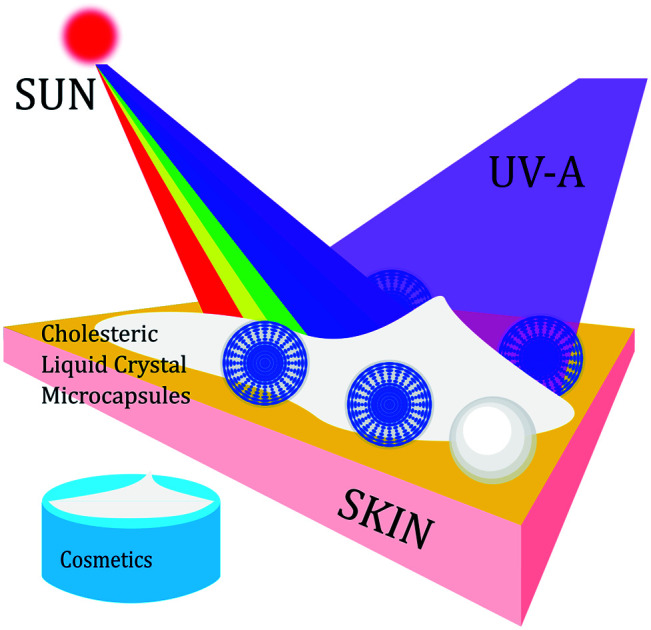

## Introduction

Recently, many people have become concerned about skin care. In particular, it is a very important issue to protect the skin against ultraviolet (UV) light from sunlight as the ozone layer of the atmosphere becomes thinner. Sunscreen can protect human skin from sunlight by decreasing exposure to UV light, specifically UV-B and UV-A. UV-B is defined as the region with an electromagnetic wavelength from 290 nm to 320 nm and UV-A is defined as the region with an electromagnetic wavelength from 320 nm to 400 nm.^1^ Each UV band has many adverse effects on the human skin, such as causing inflammation. The difference between UV-A and UV-B is that UV-A affects the dermis more than UV-B while UV-B affects the epidermis more than UV-A.^2^ UV-B causes changes in the epidermis, primarily causing sunburn of cells. More UV-B radiation is also associated with increased skin cancer.^3^ The first commercial sunscreen was developed in the 1930s. It mainly blocked radiation of the UV-B region. In 1970, sunscreen that could block both UV-A and UV-B was developed.^4^

There are two categories of sunscreen materials. One is inorganic sunscreen generally known as physical sunscreen. The other is organic sunscreen referred to as a chemical sunscreen. Each sunscreen material has its own advantages and disadvantages. Titanium dioxide (TiO_2_) is one of the main inorganic materials. It is commonly used in UV sunscreens because it can scatter both UV-A and UV-B in the solar spectrum.^5^ It is also commonly employed as a white pigment of makeup products in cosmetic applications. So far, the use of TiO_2_ in cosmetics has been generally considered as safe because it does not penetrate the stratum corneum of the human skin.^6^

However, there are some concerns that photocatalytic reactions can occur when TiO_2_ particles are irradiated by UV light.^7–11^ In an aqueous medium, TiO_2_ can act as a redox-reactive agent due to the generation of several reactive oxygen species including hydroxyl radicals, superoxide radical anions, and singlet oxygen.^12,13^ Although this property makes TiO_2_ a popular photocatalytic reagent in oxidative removal of organic pollutants,^13–15^ it likely represents a downside in the formulation of cosmetic and pharmaceutical products. To circumvent this intrinsic reactivity, many TiO_2_-based cosmetic products on the market use surface-coated particles with polymer to ensure that human skin does not have a direct contact with the redox-reactive surface of TiO_2_.^16^ Nonetheless, the stability of these coatings is not always reliable. Complete quenching of TiO_2_ reactivity cannot be ensured for a long period of time.^9,17–19^

TiO_2_ is less effective in protecting skin against UV-A compared to UV-B. In 2007, the U.S. Food and Drug Administration revealed the importance of UV-A blocking ability of sunscreens.^20^ Among the energies of UV radiation at the earth's surface, UV-A accounts for 95%, while UV-B accounts for only 5%.^21^ Therefore, although UV-B blocking is important, UV-A blocking is more important to increase the efficiency of sunscreen.^22^ Avobenzone is a globally approved UV-A sunscreen ingredient. It can provide a very good coverage for the UV-A region. However, it has an instability issue when it is exposed to UV radiation.^23^

In this study, we propose a new type of UV-A screen system on the basis of selective reflection of UV using cholesteric liquid crystal (CLC) having a periodic helical twist chiral pitch. The CLC exhibits a twisting of the molecules perpendicular to the director, with the molecular axis parallel to the director. The chirality of CLC is usually specified by helical twist chiral pitch (*P*), the distance over which LC molecules undergo a full 360° twist. It corresponds to selective wavelength (*λ*) reflection against incident light based on Bragg's reflection law of *λ* = *nP* (*n* is refractive index). To achieve our goal to reflect UV-A using CLC mixture for cosmetic application, several things need to be considered. First, compounds to be used should be listed in the international cosmetics ingredient dictionary (ICID) for preventing safety issues. Second, they should reflect UV-A light within the skin temperature range of 32–36 °C. Third, materials need to keep their optical properties when they are mixed with other cosmetic materials. This implies that the CLC mixture should be surrounded by a polymer capsule to stably maintain its intrinsic chiral pitch in the mixture since other ingredients such as oil, water, and surfactant will be mixed with the CLC mixture in a UV sunscreen emulsion. Therefore, capsules of several tens of micrometers including CLC mixture with an appropriate helical chiral pitch were used to selectively reflect wavelengths of UV light.

## Results and discussion

### Phase transition behavior and reflection wavelength of CLC mixture

**Fig. 1:**
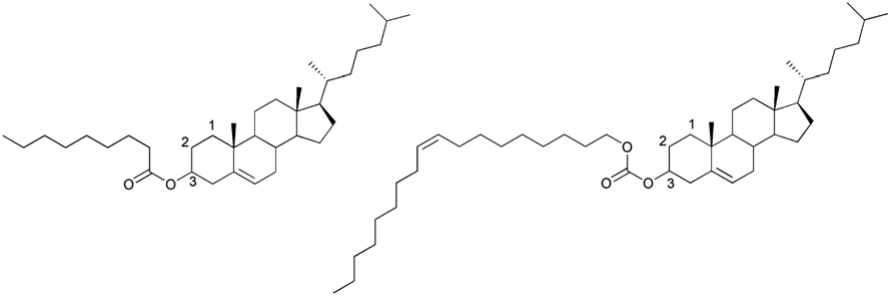
Chemical structures of cholesteric liquid crystal used in this study. Left: cholesteryl nonanoate (CN); right: cholesteryl oleyl carbonate (COC).

The liquid crystal compounds used were cholesteryl nonanoate (CN) and cholesteryl oleyl carbonate (COC) (Fig. 1). The phase transition behavior of CLC mixture is very important to determine the usage temperature for application as a UV screen because the CLC mixture only exhibits selective reflection characteristic at a cholesteric phase.

**Fig. 2:**
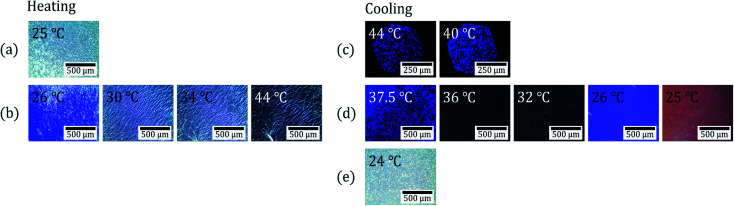
POM observation images of a CLC mixture (COC : CN = 80 : 20) at various temperatures under crossed Nicols. (a) The crystal phase at 25 °C. (b) The cholesteric phases on heating. (c) The blue phases on cooling. (d) The cholesteric phases on cooling. (e) The crystal phase at 24 °C.

Table 1 shows the phase transition temperatures for four types of CLC mixtures observed by a polarizing optical microscope (POM) on heating and cooling (Fig. 2). Upon heating, all four CLC mixtures showed three phases: an isotropic (iso) phase, a cholesteric (N*) phase, and a crystal (K) phase. However, upon cooling, they showed four phases: isotropic phase, blue phase I (BP-I), cholesteric phase, and crystal phase. Here the blue phase (BP) is a special case of CLC phase and can appear in a temperature range between N* and iso phase under high chirality (eq. short chiral pitch). BP exhibits three phases, blue phase I (BP-I), blue phase II (BP-II) and blue phase-III (BP-III), as functions of temperature and chirality.^24–26^ BP-I of Table 1 was confirmed by observing platelet texture (which is the intrinsic optical texture of BP-I; see Fig. 2(c)) using POM.

**Table 1:** Phase transition temperatures of four types of CLC mixtures observed by POM upon heating and cooling

Sample composition ratio (wt%)	Heating (°C)		Cooling (°C)		
(COC : CN)	*T*_K→N*_	*T*_N*→iso_	*T*_iso→BP-I_	*T*_BP→N*_	*T*_N*→K_
100 : 0	20.9	33.9	33.9	27.9	21.0
90 : 10	23.2	39.9	39.2	33.1	22.2
80 : 20	25.5	44.9	44.5	38.1	24.2
70 : 30	29.0	49.3	49.0	33.2	24.1

**Fig. 3:**
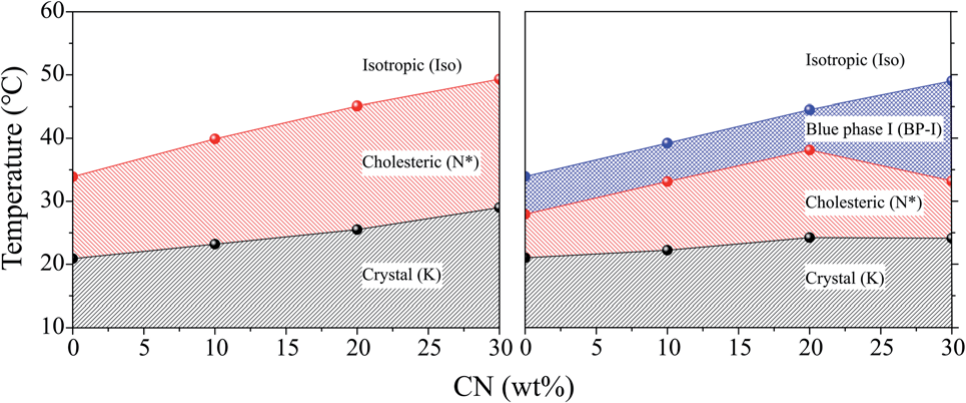
Phase diagrams for four types of CLC mixtures upon heating (left) and cooling (right).

Fig. 3 shows a phase diagram for four types of CLC mixtures upon heating and cooling on the basis of phase transition temperatures shown in Table 1. For cooling and heating processes, all phase transition temperatures were the lowest for the CLC mixture at COC : CN = 100 : 0. They increased with increasing CN concentration except for T_BP→N*_ upon cooling. The CN molecule has a linear acyl chain of nine carbon atoms, but the COC molecule has a bent configuration of alkene side chain of eighteen carbon atoms on the 3-β position^27^ as shown in Fig. 1. Therefore, it is considered that the increase in the CN ratio causes the rise in the phase transition temperature and chirality of CLC mixture due to the CN molecular linearity in the (COC/CN) mixture. In general, it is known that BP-I temperature range increases with an increase of chirality of CLC molecules.^28,29^ This can support that BP-I temperature range is increased more than the ratio of (COC : CN = 80 : 20) for the cooling process. In contrast, it is considered that the thermal fluctuation of CLC molecules with increasing temperature may offset this CN molecular linearity effect for a heating process.

In order to use CLC mixtures as a UV blocking material, the CLC mixtures should exist in the cholesteric phase within the skin temperature range of 32–36 °C. Therefore, it was necessary to adjust the composition of the CLC mixture so that it had a suitable property by adding CN to COC. As shown in Table 1 and Fig. 3, it was found that the CLC mixture of (COC : CN = 80 : 20) was the most suitable one as a UV-blocking material since it commonly showed cholesteric phase over 25.5–44.9 °C upon heating and 38.1 to 24.2 °C upon cooling.

Fig. 2 shows POM observation images of a CLC mixture (COC : CN = 80 : 20) upon heating and cooling under crossed Nicols. The first row (Fig. 2(a)) shows the crystalline phase (K) image of the CLC mixture at 25 °C before heating. The second row (Fig. 2(b)) shows the cholesteric phase (N*) image presenting oily streak textures at 26–44 °C upon heating. The third row (Fig. 2(c)) shows the blue phase I (BP-I) image presenting platelet texture at 44 to 40 °C upon cooling. The fourth row (Fig. 2(d)) shows a cholesteric phase (N*) image at 37.5 to 25 °C upon cooling. The fifth row (Fig. 2(e)) is the crystalline phase (K) image at 24 °C upon cooling. These results indicate that the CLC mixture (COC : CN = 80 : 20) presents cholesteric phase in the temperature range of the human skin (32–36 °C) upon cooling and heating.

**Fig. 4:**
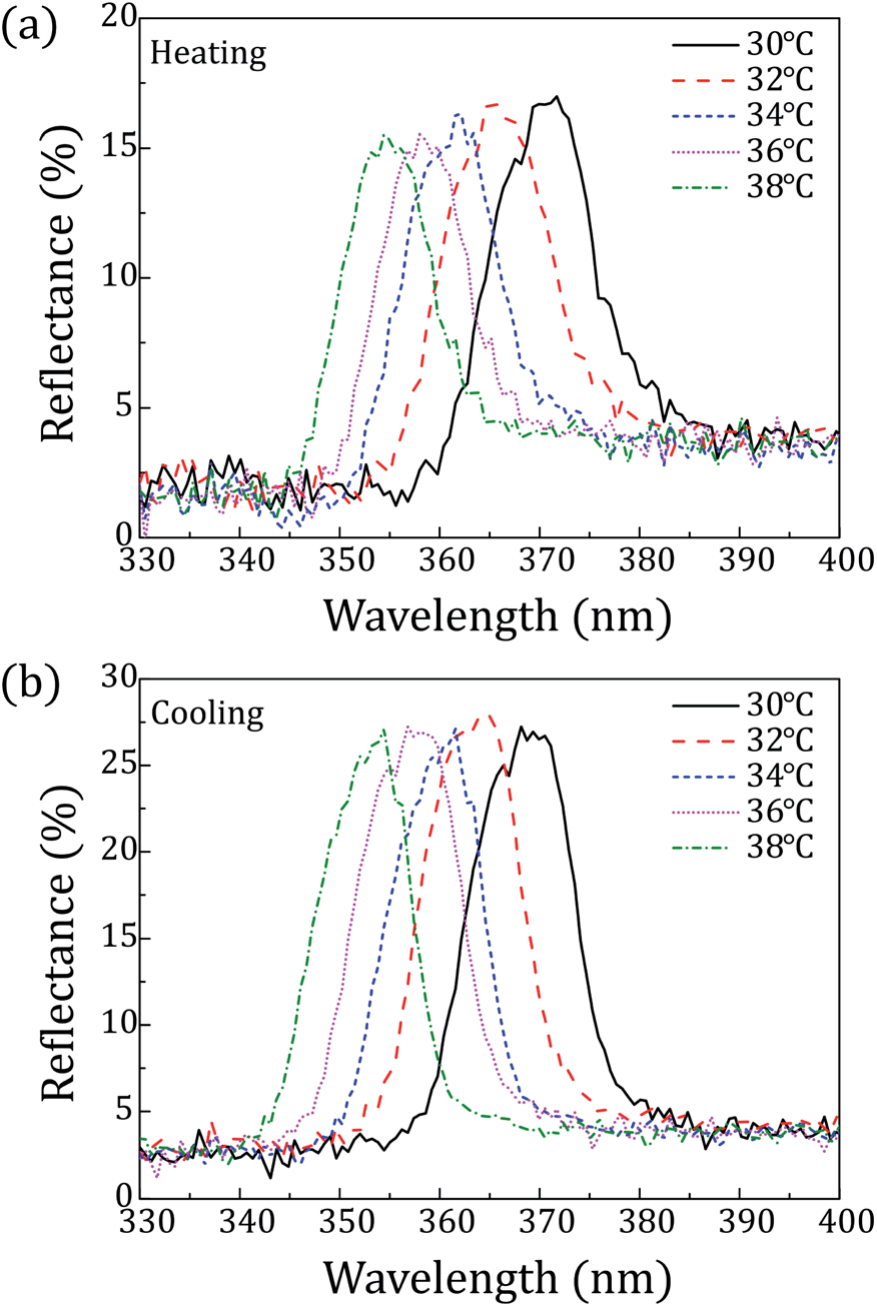
Reflection spectra of the CLC mixture (COC : CN = 80 : 20) depending on the temperature: (a) heating; (b) cooling.

Fig. 4 shows reflection spectra of the CLC mixture at each temperature. All reflection spectra shifted to shorter wavelength with increasing temperature. This is because this CLC mixture has a left-handed helical structure whose chiral pitch is inversely proportional to temperature.^30^

Peaks of reflection wavelength appeared in the range from 366 nm to 358 nm upon heating and in the range from 357 nm to 364 nm upon cooling over 32–36 °C. Thus, the CLC mixture (COC : CN = 80 : 20) can commonly cover UV light over 358–364 nm within the skin temperature range. Furthermore, considering peak surrounding, it can be said that reflection spectra approximately cover the wavelength range of 350 nm to 380 nm over 32–36 °C as shown in Fig. 4.

### The appearance of CLC/polycaprolactone (PCL) microcapsules

**Fig. 5:**
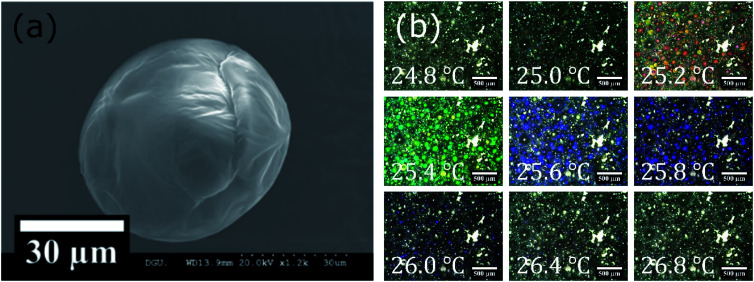
(a) SEM and (b) optical microscope images of microcapsules prepared with 1% (w/v) PVA solution.

Size distributions and encapsulation yields of CLC/PLC microcapsules including CLC mixture (COC : CN = 80 : 20) depending on the concentration of the stabilizer (poly(vinyl alcohol) (PVA) solution) are listed in the ESI† (Table S1 and Fig. S1). Fig. 5(a) shows a scanning electron microscope (SEM) observation image of a CLC/PCL microcapsule prepared with 1% (w/v) PVA solution. The SEM image showed that the microcapsule had a diameter of roughly 50–60 μm with a smooth surface. For 1% (w/v) PVA solution, the average diameter and median were 34.2 and 25.4 μm respectively. The smooth surface of the capsule offers homogeneous optical property and the ability to prevent unwanted light scattering. The reflection colour was observed as red at 25.2 °C, green at 25.4 °C, blue at 25.6 °C, violet at 25.8 °C, and no colour at 26.4 °C as shown in optical microscope images (Fig. 5(b)). The reason was that the chiral pitch of the CLC mixture decreased with an increase in temperature. The CLC mixture began to reflect UV-A rather than visible light when the temperature exceeded 26.4 °C.

**Fig. 6:**
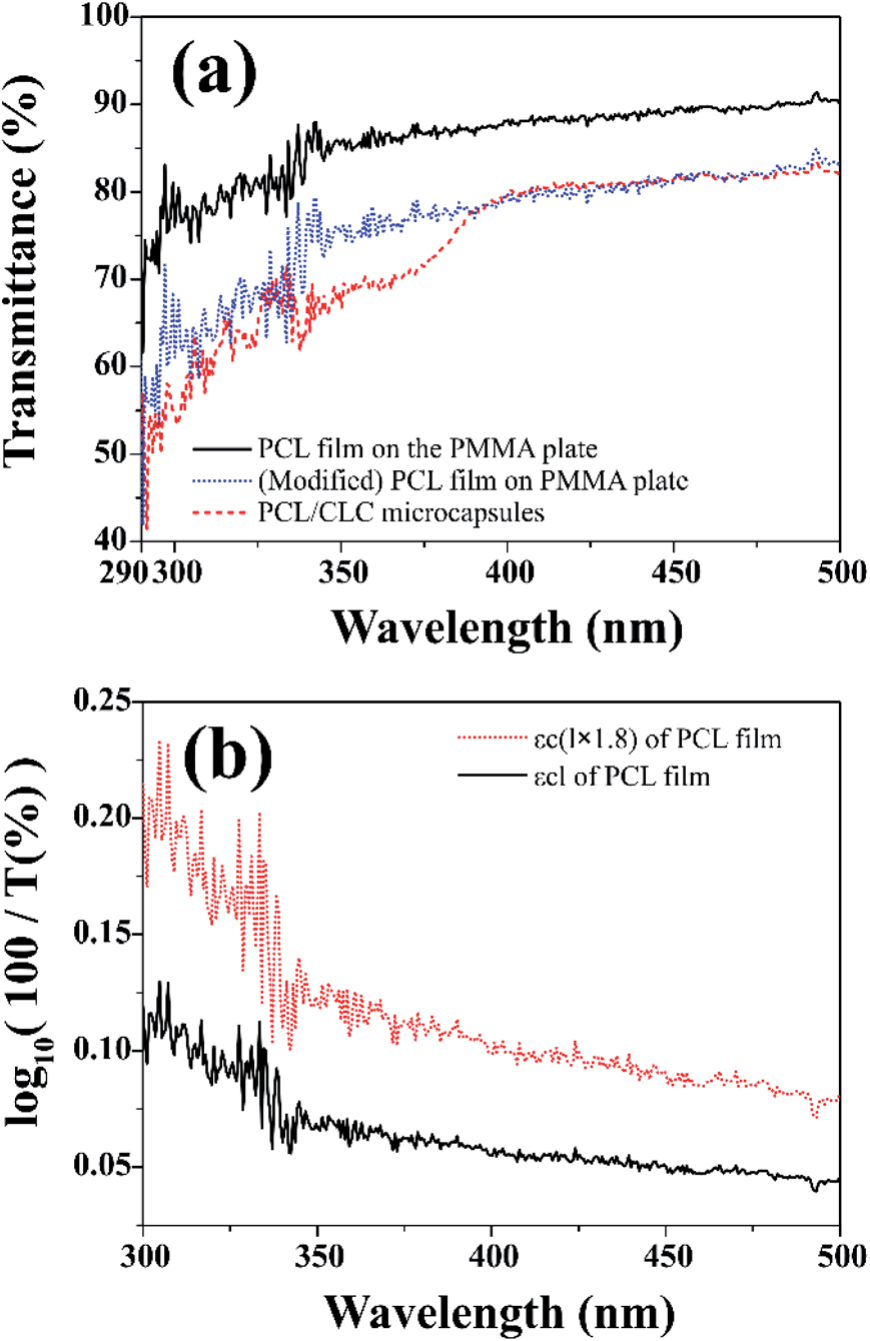
(a) (black solid line) Transmission spectrum of PCL film coated on a PMMA plate. (blue dotted line) Modified transmission spectrum of PCL film coated on a PMMA plate. (red dashed line) Transmission spectrum of CLC/PCL microcapsules on a PMMA plate measured at 32 °C. (b) (black solid line) A plot of *εcl* calculated from the transmission spectrum of PCL film depending on wavelength. (red dotted line) A plot of 1.8 times *εcl* of PCL film depending on wavelength.

Fig. 6(a) shows UV-visible transmission spectra of PCL film and CLC/PCL microcapsules over 290–500 nm. UV blocking ability was evaluated through the transmission spectrum of CLC/PCL microcapsules coated on a transparent PMMA plate on the basis of measurement of *in vitro* SPF values commonly used in the cosmetics industry.^31^

It is known that *in vitro* SPF can be measured using the UV-visible spectrum transmitted through the sunscreen and PMMA plate when a specific amount of sunscreen is applied on the PMMA. The measured transmission spectrum of a single PCL film on PMMA was used as a baseline to compare with that of CLC/PCL microcapsules for evaluating UV blocking ability. However, the measured transmission spectrum of the PCL film did not overlap with that of the CLC/PCL microcapsules in all wavelength ranges as shown in Fig. 6(a). This might be due to the fact that the optical path length of the PCL film differs from that of the CLC/PCL microcapsules due to thickness difference between a PCL film and a PCL shell of CLC/PCL microcapsules or light scattering. Therefore, we tried to mathematically modify the transmission spectrum of the PCL film using eqn (1) (Beer's law) and eqn (2) to overlap with that of CLC/PCL microcapsules:1
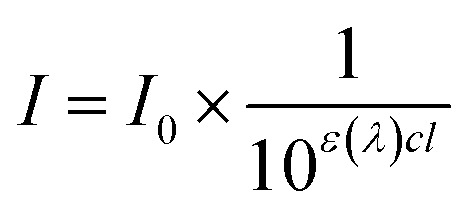
2
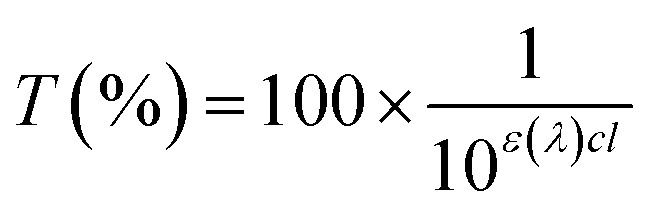
where: *I*_0_: incident light intensity; *I*: transmitted light intensity; *T*: transmittance; *ε*(*λ*): molar extinction coefficient (M^−1^ cm^−1^); *c*: concentration (mol l^−1^); *l*: optical path length (cm) 3
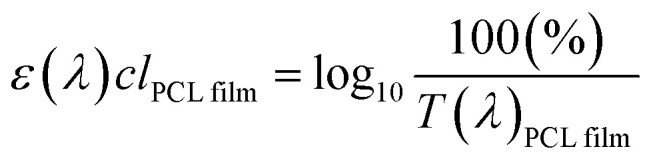

4 *ε*(*λ*)*cl*_modified PCL film_ = *ε*(*λ*)*c*(*l*_PCL film_ × 1.8)

5
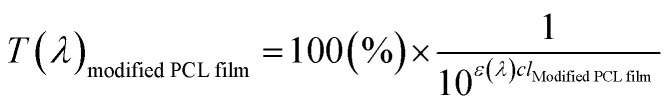


The value of *ε*(*λ*)*cl*_PCL film_ was estimated from the measured transmission spectrum of the PCL film (black solid line in Fig. 6(a)) using eqn (3) shown as a black solid line in Fig. 6(b). We empirically found that the transmittance spectrum of PCL film overlapped with that of CLC/PCL microcapsules through introduction of *ε*(*λ*)*cl*_modified PCL film_ multiplied by 1.8 times to *ε*(*λ*)*cl*_PCL film_ shown as a red dotted line in Fig. 6(b). This implies that the optical path length of the PCL shell of a microcapsule is increased by 1.8 times compared to that for a single PCL film. *T*(*λ*)_modified PCL film_ was then recalculated using eqn (5) and plotted as a blue dotted line shown in Fig. 6(a). Eqn (6) was defined to evaluate the UV blocking ability of CLC/PCL microcapsules by comparing transmittance of PLC film and CLC/PCL microcapsule over the wavelength range 290–400 nm:6
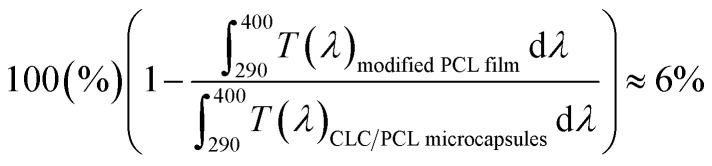


As a result, the integrated transmittance of CLC/PCL microcapsules was reduced by about 6% in the range of 290–400 nm compared to the transmittance of the modified PCL film.

This indicates that the CLC/PCL microcapsules can reflect about 6% UV in the wavelength range of 290 to 400 nm at 32 °C. Furthermore, we can know that CLC/PCL microcapsules mainly reflect UV-A in the range of 350–380 nm similar to the CLC mixture. However, its reflectivity is smaller than the CLC mixture as shown in Fig. 4 and 6(a).

We tried to reason why the UV reflectivity of the CLC/PCL microcapsules was lower than that of the CLC mixture (COC : CN = 80 : 20). The first reason was that the translucent property of PCL might have interfered with the selective reflection by CLC. The second reason was that it was difficult for microcapsules to be coated closely and uniformly on all sides of the PMMA plate.

## Experimental

### Preparation of CLC mixture

Fig. 1 shows the chemical structures of cholesteric liquid crystals used in this study. Two kinds of cholesteric single compounds, CN (97%, Sigma-Aldrich) and COC (reagent grade, Sigma-Aldrich), were used as suitable CLC single materials to block UV light in the skin temperature range. For the encapsulation of CLC surrounded by polymer, PCL (*M*_n_ = 45 000) as a polymer shell, dichloromethane (DCM) as a solvent (99.5%, Samchun Chemical), and PVA (*M*_n_ ∼ 2000, degree of saponification *ca.* 80 mol%, Tokyo Chemical Industry) as a stabilizer were used. COC, CN, PCL, and PVA are listed on ICID. Furthermore, four types of CLC mixtures composed of COC and CN were prepared in terms of weight percent as follows: COC : CN = 100 : 0, COC : CN = 90 : 10, COC : CN = 80 : 20, and COC : CN = 70 : 30.

### Preparation of CLC/PCL microcapsules

**Fig. 7:**
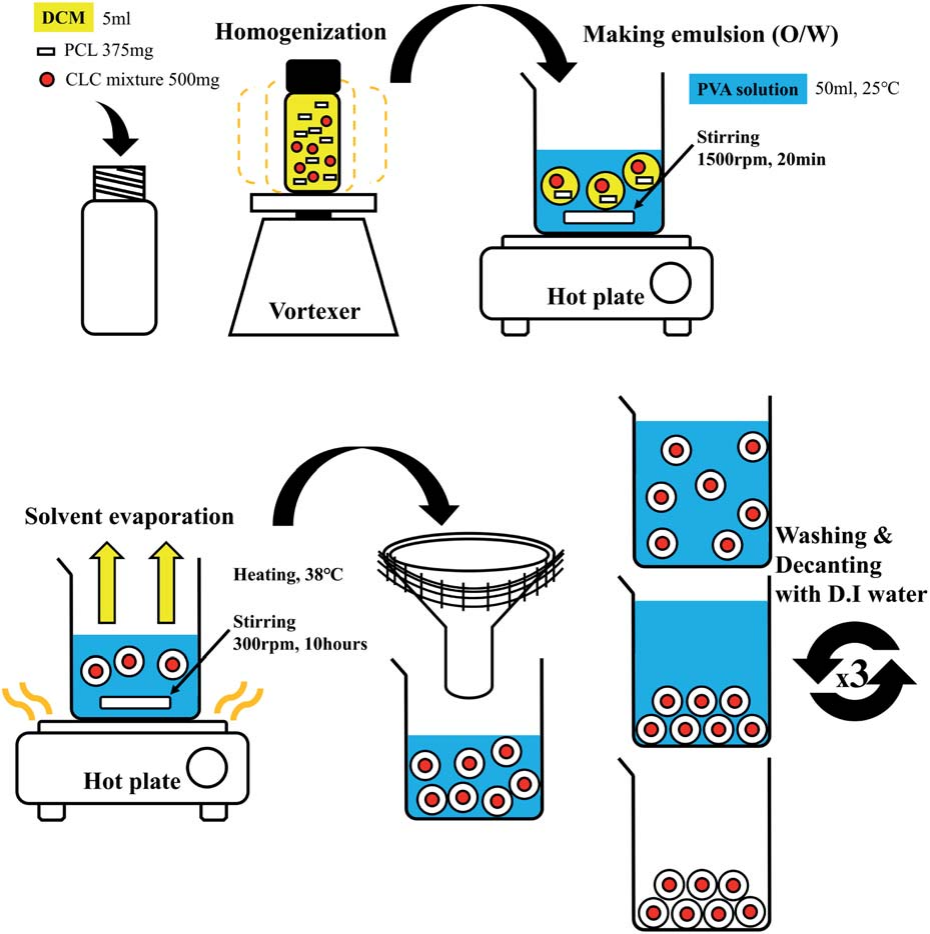
Schematic representation of fabrication process of CLC/PCL microcapsules.

Fig. 7 shows a schematic representation of the fabrication process of CLC microcapsules surrounded by PCL. PCL of 375 mg and CLC mixture of 500 mg were completely dissolved in DCM (5 ml). Then 1% (w/v), 2% (w/v), and 3% (w/v) PVA aqueous solution (50 ml) was added to the CLC/PCL solution and stirred at 1500 rpm with a magnetic stirring bar at room temperature. After 20 min, the agitation rate was reduced to 300 rpm and more PVA aqueous solution (50 ml, 25 °C) was added into the same beaker. The solution was heated at 37 °C for 10 h to evaporate the DCM solvent. After the solvent was evaporated, PCL and CLC mixture was transformed to microcapsules with shell (PCL) and core (CLC mixture) materials being separated. These microcapsules were washed with DI water and decanted repeatedly more than three times. Thereafter, these microcapsules were dried in a vacuum desiccator at room temperature.^32^

### Characterization of CLC mixtures and CLC/PCL microcapsules

Reflection spectra were obtained with a fiber optic spectrometer (Avaspec-2048L, Avantes) in reflection mode at normal incidence (see ESI Fig. S2†). The intrinsic reflection spectrum of the CLC mixture was measured over a temperature range of 27 °C to 44 °C using a glass cell. The temperature of each cell was controlled at 1.0 °C min^−1^ on heating and cooling with a hot stage (LTS 420, Linkam).

Transmission spectra of CLC/PCL microcapsules were obtained with a light source (DC xenon arc lamp, model 10500, ABET Technologies), an integrating sphere, a fiber optic spectrometer, and a standard PMMA plate (8-5105 molded PMMA plates, solar light). A PMMA plate is commonly used for *in vitro* SPF measurements and is also ISO 24443 standard (see ESI Fig. S3†). Each sample was measured at 32 °C.

A POM (Eclipse LV100 POL, Nikon) equipped with a digital camera (G1UD03C, JPLY) was used for defining the phase of the CLC mixture under crossed Nicols. All CLC mixtures were controlled at 1 °C min^−1^ over a temperature range of 24 °C to 44 °C with a hot stage. The surface morphology of the microcapsules was observed with a SEM (Hitachi S-3000N, Hitachi Instruments) at room temperature.

## Conclusions

We confirmed that CLC mixtures could reflect UV-A over 350–380 nm within the human skin temperature range (32–36 °C) when the composition of the CLC was COC : CN = 80 : 20. The CLC/PCL microcapsules with average diameter of 34.2 μm were prepared to keep the intrinsic helical chiral pitch corresponding to the wavelength of UV light made by a solvent evaporation method. This study also confirmed that CLC/PCL microcapsules could block UV light over 290–400 nm by about 6%. Therefore, CLC mixture and CLC/PCL microcapsules could be used as a new type of UV sunscreen.

## Conflicts of interest

There are no conflicts to declare.

## Supplementary Material

RA-011-D1RA03499E-s001
