# Supplementary material for: Ultraviolet light screen using cholesteric liquid crystal capsules on the basis of selective reflection
Source: RSC Adv. 2021 Jul 22;11(41):25471–6. doi: 10.1039/d1ra03499e (PMC9037046; doi:10.1039/d1ra03499e)
Supplement: RA-011-D1RA03499E-s001 [file RA-011-D1RA03499E-s001.pdf]

Electronic Supplementary Material (ESI) for RSC Advances.
This journal is © The Royal Society of Chemistry 2021

(Supporting Information)

Table S1 and Figure S1 show particle size distributions and capsule yields of CLC/PCL microcapsules with varying concentration of stabilizer PVA. For 1 (w/v)% PVA solution, the average diameter and yield were 34.2 μm and 50%, for 2 (w/v)% PVA solution were 151.8 μm and 50%, and for 3 (w/v)% PVA solution were 115.2 μm and 70%, respectively. As a result, 1 (w/v)% PVA solution showed the appropriate value in terms of yield and size among the three series of conditions because several tens of micrometer capsule is applicable for cosmetic applications. The sizes and distributions were analysed using ImageJ software. The yield for capsules were defined as the ratio of obtained microcapsule mass to used shell PCL and core materials CLC mixture mass.

Table S1 Particle characteristics of PCL/CLC microcapsules by PVA solution concentrations

	Average(μm)	Median(μm)	Yield (%)[a]
3 (w/v)% PVA solution	115.2	102.6	70
2 (w/v)% PVA solution	151.8	147.7	50
1 (w/v)% PVA solution	34.2	25.4	50

[a] The yield was obtained from the conversion measurements. ($\frac{MASS\ of\ obtained\ microcapsules}{MASS\ of\ used\ shell\ and\ core\ materials} \times 100(\%) = Yield$)

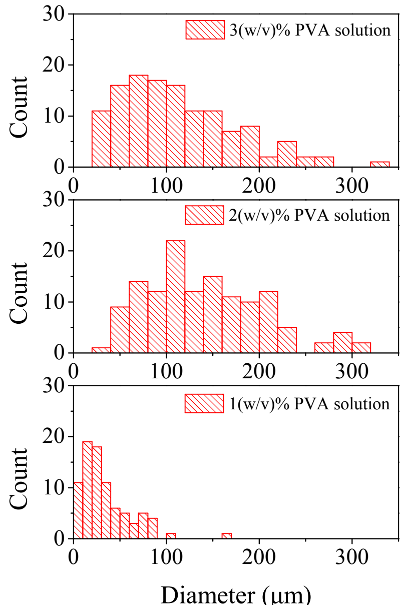

Figure S1 Particle size distribution of (PCL/CLC) capsules

Figure S2 shows the schematic representation of experimental set up with a fiber optic spectrometer in reflection mode at normal incident to measure the UV-Vis spectrum of the CLC mixture on the glass cell.

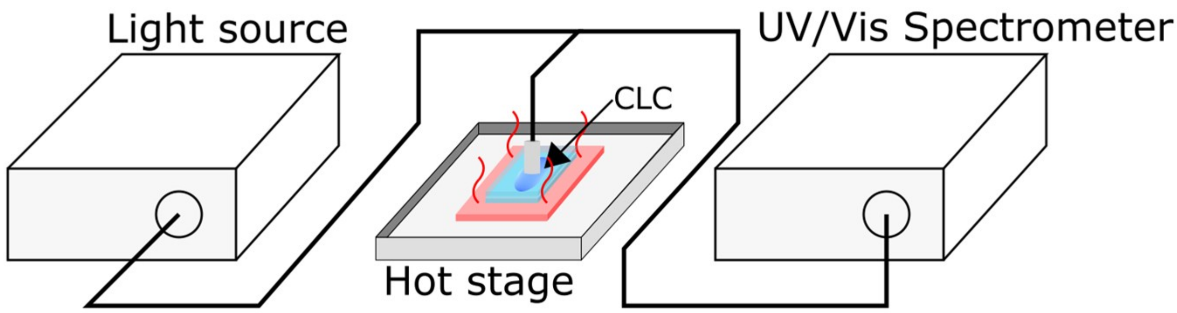

Figure S2 Schematic representation of experimental set up to measure the UV-Vis reflective spectrum of CLC mixture.

Figure S3 shows the photograph of experimental set up to measure the UV-Vis transmission spectrum of CLC/PCL microcapsules. This set up is composed of a light source (ABET technologies) from solar simulator, a integrating sphere, a fiber optic spectrometer (AvaSpec-2048L, Avantes) and a standard PMMA plate (8-5105 molded

PMMA plates, Solar light). The PMMA plate is commonly used for *in vitro* SPF measurements and is also ISO24443 standard.

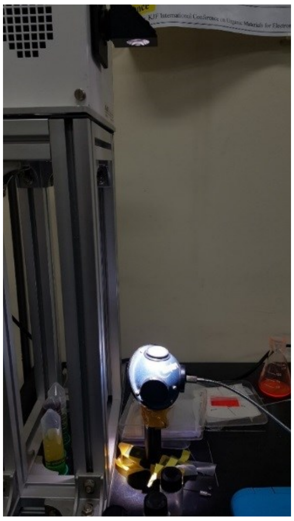

Figure S3 Experimental set up to measure the UV-Vis transmission spectrum of CLC/PCL microcapsules.
